# The Effect of Active Stretching Training in Patients with Chronic Venous Insufficiency Monitored by Raster-Stereography

**DOI:** 10.3390/s22218509

**Published:** 2022-11-04

**Authors:** Erica Menegatti, Simona Mandini, Anselmo Pagani, Beatrice Mandini, Valentina Zerbini, Tommaso Piva, Andrea Raisi, Marinella Fabbri, Marco Fogli, Gianni Mazzoni, Paolo Zamboni, Sergio Gianesini

**Affiliations:** 1 Department of Environmental and Prevention Sciences, University of Ferrara, 44123 Ferrara, Italy; 2 Center for Exercise Science and Sport, Department of Neuroscience and Rehabilitation, University of Ferrara, 44123 Ferrara, Italy; 3 Vascular Diseases Center, Department of Translational Medicine, University of Ferrara, 44124 Ferrara, Italy; 4 Department of Surgery, Uniformed Services University of the Health Sciences, Bethesda, MD 20814, USA

**Keywords:** raster-stereography, posture, gait, chronic venous disease, oedema, exercise therapy

## Abstract

(1) Background: Musculoskeletal disorders can be associated with advanced clinical stages of chronic venous insufficiency (CVI). The aim of the study is to investigate the effect of active stretching (AS) training on lower limb venous function and quality of life in patients affected by CVI. (2) Methods: A prospective two-armed pilot randomized controlled was conducted. Twenty (20) CVI patients were randomly assigned to an AS training or to a control group (C) who did not receive any exercise indication. At baseline and after three months all the participants were tested for leg volumetry (LV), air plethysmography (APG), and quality of life (QoL) measured by a disease specific validated questionnaire (VVSymQ), ankle range of motion (ROM), and postural deformities using an optoelectronic body posture machine. (3) Results: At the end of the training in the AS group a significant leg volume reduction was detected (from 2340 ± 239 mL to 2239 ± 237 mL (4.3%); *p* < 0.0001), whereas in the C group no significant volume changes were found. The ejection fraction rate (EF%) increased significantly from 49.3 ± 9.3 to 61.1 ± 14.5, *p* < 0.005. A moderate-strong linear correlation with EF% and ankle ROM variation was found (R^2^ = 0.6790; *p* < 0.0034). Several postural outcomes such as pelvic tilt, pelvic torsion, and lordotic angle significantly improved in the AS group (*p* < 0.01, *p* < 0.04, *p* < 0.01 respectively). (4) Conclusion: The AS training impacts on the APG parameters related to the musculoskeletal pump efficiency, opening a further possibility in the management of CVI patients by means of an appropriate adapted physical exercise program.

## 1. Introduction

Chronic venous insufficiency (CVI) is an extremely common vascular pathology affecting up to 50% of the general population in industrialized countries [1,2].

The clinical presentation varies from aesthetic to severe manifestations, including telangiectasis, reticular veins, varicose veins, edema, pigmentation, lipodermatosclerosis, and venous ulceration [3].

The appearance and progression of CVI depend on different risk factors, such as genetic background, female sex, hormones, obesity and a sedentary lifestyle [4,5,6,7].

Another significant determinant is found in occupational and prolonged time standing or sitting, with lack of prevention potentially being one of the main causes of the disease’s progression [8].

Musculoskeletal disorders are often associated with advanced clinical stages of CVI, especially foot static disorders which in turn can also affect the body posture [9]. All the above-mentioned conditions may benefit from adapted physical exercise (APE) requiring standardized protocols. A recent systematic review evaluated the effects of APE on clinical signs, symptoms, and quality of life (QoL) in chronic venous disease (CVD), confirming their usefulness as complementary therapeutic options for the care of CVD patients [10].

Most of the literature regarding the effect of exercise in CVD include studies in aquatic environment, both in normal and in thermal water. The first studies considered outcomes related to quality of life (QoL) and symptoms, and mostly report non-standard exercise protocols such as walking in the pool without precise indication [11,12,13,14]. On the other hand, a different group of studies investigating the effect of exercise was performed on dry land with a focus mainly on QoL [15,16,17].

In 2021, two prospective randomized controlled trials were performed, one in a thermal aquatic environment [18] and one on dry land [19]. These trials report the clinical outcome together with QoL, demonstrating the benefit of APE in patients with CVI in terms of leg volume decrease and QoL improvement respect to a control group. The exercise protocol performed in both cases was standardized and supervised but, on dry land, the intervention group was combined with compression therapy. The use of compression therapy can change transmular pressure (differential pressure inside and outside the venous wall), increasing external venous pressure [20] and so contribute to the improvement of venous function and symptomatology in CVI. This point represents a bias in assessing the effect of APE in CVI as a standalone treatment.

During the early stage of the COVID-19 pandemic a combination of containment and mitigation strategies were implemented worldwide. While these strategies are imperative to public health, they may lead to unintended consequences for health, including excessive physical inactivity (PI) and sedentary behavior (SB) [21,22]. In order to reduce PI and SB, it is therefore necessary to take into consideration the promotion strategies that can be delivered in the presence of restrictions due to the COVID-19 pandemic, which otherwise would not allow supervised motor activity. Tele-exercise, using direct supervision with the use of webcams, telephone calls or smartphone applications, has produced comparable improvements in outcomes compared with clinic or hospital-based, in-person, control interventions in subjects with musculoskeletal conditions and chronic disease [23].

For this reason, the aim of this study is to investigate the effect of tele-monitored active stretching training (AS) on venous function and quality of life in patients with CVI.

The secondary objective is to assess the presence of postural deformities associated to CVI and the changes in postural outcomes following AS intervention measured by optoelectronic body posture machine

## 2. Materials and Methods

### 2.1. Study Design and Setting

This prospective single-blinded randomized controlled trial with two parallel arms was conducted from May 2021 to September 2021. All the patients were evaluated at the beginning of the program (T0) and after three months (T3) at the Vascular Disease Center at the University of Ferrara (Italy). The intervention group was followed online using the Google Meet platform by two expert exercise physiologists. The video-conferencing equipment was developed to ensure that interactions between exercise physiologist and patients during the tele-exercise sessions were not impeded by technology.

### 2.2. Study Population

Twenty-five (25) consecutive subjects (6M/19F; average age 58.9 ± 12.7), referred to the Vascular Disease Center, University of Ferrara (Italy) for CVI were considered eligible for the enrollment. The inclusion criteria were:

- Age > 18 years
- Bilateral chronic venous insufficiency CEAP clinical classification [3]: C2-3EpAsPr.

The exclusion criteria were:

- Previous lower limbs trauma
- Heart/respiratory/renal insufficiency
- Use of graduated compression stockings
- Competitive physical activity
- Physical activity in the aquatic environment
- Body mass index > 35
- Inability to perform any form of physical activity

### 2.3. Randomization

The randomization list was generated using dedicated software (https://wwwservizi.regione.emilia-romagna.it/generatore/, accessed on 8 October 2022), the cohort was allocated in 1:1 fashion to a structured AS or to control group (C) where the patient did not perform any type of exercise.

### 2.4. Intervention

**Figure 1:**
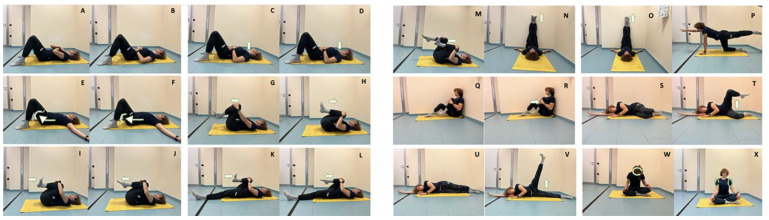
Example of active stretching exercise protocol (the letters(**A**–**X**) are referred to each exercise reported in Table 1).

The AS exercise program was administered and supervised by an exercise science specialist for three consecutive months, each exercise session lasted 40 min (from 7 pm to 7:40 pm) and was repeated twice a week. The exercises were performed entirely on the floor using a mat and a free wall was required to perform the exercises “with legs up”. The exercise series started lying down on the floor in a supine position with the legs bent, subsequently the legs were stretched over the head with the request to perform a wide flexion-extension of the ankle coordinated with thoracic pump activation. All the detailed exercise series are reported in Table 1 and Figure 1.

### 2.5. Outcome Measurements

All data were collected between 10am and noon, at the beginning of the program (T0) and after three months (T3). All the clinical outcomes (leg volume, air plethysmography and ankle range of motion) were assessed both on the left and right leg.

### 2.6. Lower Limb Volumetry

Leg volume was assessed according to the Kuhnke formula (Vlimb = X2/π), using a centimeter tape [24].

Starting from the malleolus to the joint line of the knee, the leg was divided into eight sectors every 4 cm, scoring small notches with an indelible marker. The circumferences detected in each of the eight points were reported in a report sheet.

### 2.7. Air Plethysmography (APG)

The venous volumes were studied by means of APG test (ACI medical device, San Diego, CA, USA), which was performed according to the protocol described by Christopoulos et al. [25]. The test started with the subject lying down on the bed with the leg under study wearing a cuff connected to the computer and resting on a support and slightly bent outwards. When the test started, the operator quickly elevated the leg, in order to empty the venous system, subsequently, the subject was invited to stand up, putting the body load on the contralateral leg. When the venous system had filled completely and the curve on the computer screen reached a plateau, the patient was requested to stand on their tiptoes once, and then a further consecutive 10 times. The following parameters were recorded: the venous volume (VV), venous filling index (VFI) corresponding to the time require to fill 90% of VV, ejection fraction (EF%) corresponding to the venous volume fraction ejected in a single tip-toe, and RV fraction (RVF%) corresponding to the residual venous volume following 10 consecutive tiptoe stands.

### 2.8. Ankle Range of Motion (ROM)

Ankle ROM was assessed with a professional goniometer at the beginning (T0) and at the end of the exercise program (T3). The measurement was taken with the patient seated on a bed high enough to maintain the foot sole parallel to the ground while suspended and the patient was asked to make the maximum voluntary dorsiflexion and plantarflexion, [26,27] the ankle ROM was then calculated as the sum of that dorsiflexion and plantarflexion.

### 2.9. Quality of Life (QoL)

QoL was measured by disease specific questionnaire VVSymQ at baseline and at three months follow-up. This questionnaire is addressed to evaluate the symptomatology related to venous diseases; higher scores mean worse situations whereas lower score is related to lighter symptoms [28].

### 2.10. Optoelectronic Body Posture Machine

Body posture was evaluated by the optoelectronic test of body posture, the Formetric Diers 4D, using raster-stereography (DIERS Medical Systems, Chicago, IL, USA) according to the protocol reported by the manufacturer’s guidelines [29].

This examination allows one to make a non-invasive optical detection (since it does not use X-rays) of the spine and pelvis. The investigation is carried out by an instrument constituted by a projector emitting a halogen light beam toward the subject’s back. This latter reflects the light that is detected by a digital camera and analyzed by the software. The software processes the information in real time, reconstructing the tridimensional image of the spine and pelvis.

The patients were evaluated in standing position, bare-chest and turning their back to the light-emitting unit, the room was kept dark in order to avoid any interference during the detection. Images of the surface of the back were recorded, digitized, and represented in 3D space [30,31].

The following parameters related to lumbar spine and pelvis were considered:

- Lordotic angle ITL-ILS max°: the maximum lordotic angle measured between the tangents to the surface of the thoraco-lumbar inflexion point (ITL) and the lower lumbo-sacral inflexion point (ILS).
- Pelvic tilt in degrees (°): The angle between the line connecting left dimple (DL) and right dimple (DR) and the horizontal.
- Pelvic tilt (mm): pelvic tilt refers to the difference in height of the sacral dimples (DL-DR).

### 2.11. Statistical Analysis

The statistical analyses were performed using Prism 8—v8.2.1, 2019 (GraphPad Softwere Inc. San Diego, CA, USA, 92108). The data are expressed as mean standard deviation. The Kolmogorov–Smirnov test was used to evaluate the data distribution. The demographics and clinical characteristics at baseline between the two groups were compared by means of exact Fisher test or the two-tailed Student’s *t*-test for unpaired data or a Mann–Whitney test, according to the distribution of the data. To assess intra-group differences between the baseline and the end of the study, the two-tailed Student’s *t*-test for paired data or the Wilcoxon test were used. Data relating to the two sides (right and left) were averaged to obtain a single numerical value to simplify the analysis. The correlation between the ankle ROM variation and EF% was calculated using a linear regression analysis. The level of significance was set at *p* < 0.05.

## 3. Results

### 3.1. Characteristics of the Study Population

**Figure 2:**
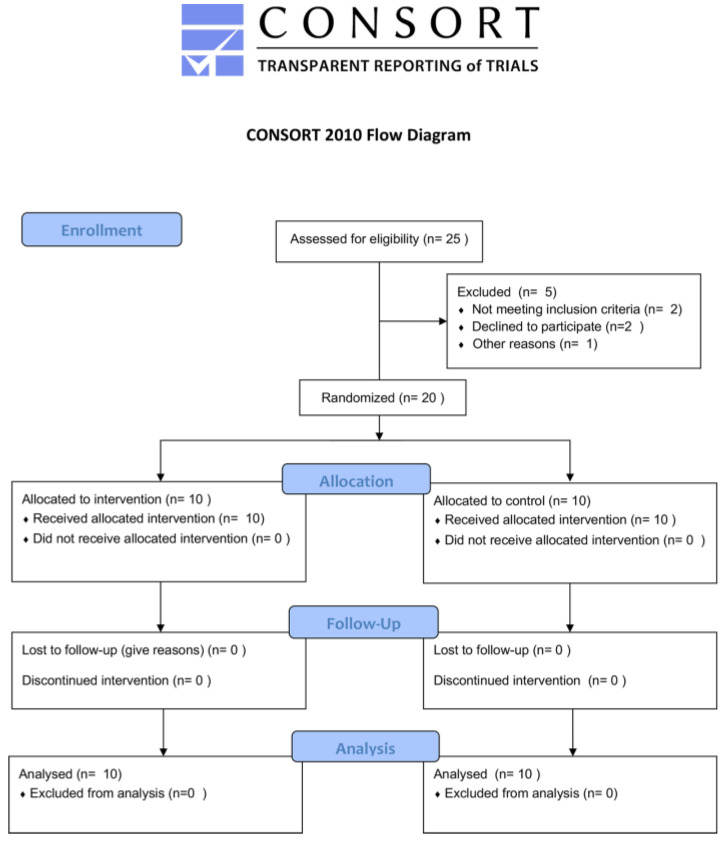
Flow diagram of the progress through the study phases according to the CONSORT 2010 statement.

Of a total of 25 eligible patients, 5 were excluded as per the specifications in Figure 1, while 20 were included and randomized in the study for a total of 40 lower limbs (Figure 2).

All patients randomized to the AS exercise group completed the protocol and no major or minor adverse events were reported. The clinical and demographic characteristics of the study participants are given in Table 2.

### 3.2. Lower Limb Volume

**Figure 3:**
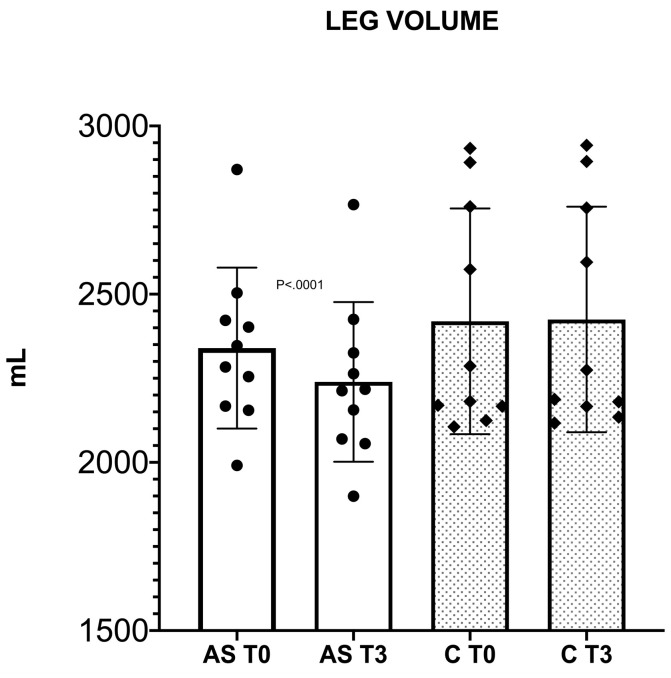
Leg volume significant reduction after three months follow-up in AS group (white histogram), while no significant changes in leg volume were recorded in the control group (dotted grey histogram).

Following three months of the AS exercise program a significant leg volume reduction was detected (from 2339.9 ± 239.2 mL to 2239.2 ± 237.2 mL (4.3%), *p* < 0.0001), whereas in the C group no significant variation was found (from 2419.2 ± 335.5 mL to 2424.8 ± 335.3 mL, *p* = 0.0917) as shown in Figure 3.

### 3.3. Air Plethysmography (APG)

In AS group at three months follow-up the EF% reported a significant increase ranging from 49.3 ± 9.3% to 61.1 ± 14.5%, *p* < 0.005. This latter is related to the efficiency of the calf muscle pump. No significant changes were detected for the other APG parameters. The C group did not show any significant variation for any of the tested APG parameters.

### 3.4. Ankle ROM

At the same observation time, in the AS exercise group the ankle ROM significantly improved from 42.7° ± 4.5 to 50.7 ± 8.3° (*p* < 0.0045), while in the control group it did not.

**Figure 4:**
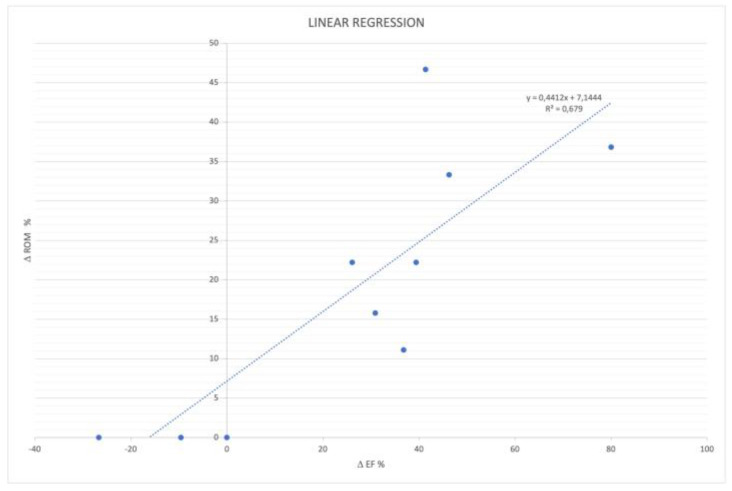
Correlation between ankle ROM and EF% variations in the AS exercise group.

Moreover, in the AS exercise group a linear correlation between the ankle ROM and EF% improvement was identified (R^2^ = 0.6790; *p* < 0.0034), as shown in Figure 4.

### 3.5. Quality of Life

At the end of the AS exercises program a significant improvement in venous symptomatology was measured, the VVSymQ total score decreased from 11.1 ± 2.7 to 3.7 ± 0.8, *p* < 0.0001; while no significant changes were recorded in the C group.

### 3.6. Body Posture Evaluation

All parameters related to the lumbar spine and pelvis significantly improved in the AS exercise group while no significant changes were detected in the C group at three months follow-up, the detailed values are reported in Table 3.

## 4. Discussion

The close relationship between the musculoskeletal system and venous return has been the subject of investigation for a long time [9].

In physiological conditions, moving from supine to standing, and under the influence of gravity, the intravenous pressure is around 90 mmHg, while during walking (after 10–20 steps) it decreases around 30 mmHg: this phenomenon is related to blood volume mobilization, due to the activation of muscular pumps [32].

In case of venous diseases, intravenous pressure and APG parameters worsen [33].

Additionally, musculoskeletal system impairment, especially calf muscle pump and plantar support disorders, are often associated with advanced clinical stages of CVI and worse APG parameters [9,34,35].

In 2016 a Cochrane review concluded that there is currently not enough evidence to assess the efficacy of physical exercise in people with CVI. Further research is encouraged especially for the assessment of physical exercise effect based on type, intensity, frequency, and timing [36].

According to our results, the proposed exercise protocol, by stimulating the active stretching of the posterior kinetic muscles chain until the plantar sole, demonstrated to be effective in improving the EF% (+24%), as measured by air plethysmography. In addition, a significant improvement of ankle ROM showed a linear correlation with EF% increase, corresponding to a better calf pump function. This phenomenon highlighted the importance of proper ankle joint mobilization to maintain the efficacy of the valvulo-muscular calf pump [37].

The postural evaluation by means of raster-stereography showed at baseline different anomalies in association with venous and lymphatic drainage impairment [28,38]. The improvement of pelvic torsion around 2° after the AS program is also in line with those already published in the literature on spine and pelvis rehabilitation programs, mostly using bone manipulations such as mobilization with movement measured by raster-stereography [39]. The strengthening of the AS program is related to the fact that the patients actively stretch their posterior muscular chain in coordination with thoracic and triceps pumps activation and that this produced an effect not only on the postural outcome, as majorly expected, but also on venous return and haemodynamics. The data herein presented support the hypothesis that postural abnormalities are present in CVI patients, with a particular involvement of the lumbar spine and pelvic region. The non-invasive raster-stereographic measuring system could potentially be used in a follow-up of exercise program addressed to spinal and pelvic abnormalities and to reducing ionizing radiation exposure.

The AS exercise program combined with a proper breathing control was also aimed at improving spine alignment and pelvis mobility. Indeed, the postural abnormalities related to lower back were significantly improved after three months.

Another important clue was the leg volume reduction following AS exercise program, which corresponds to less oedema and interstitial fluids accumulation. This phenomenon can be explained by the more efficient pump muscle function in edema removal following the adapted physical training.

The average reduction was moderate—100.6 ± 26.3 mL—but significant enough to induce a significant improvement of venous insufficiency related symptoms measured by VVSymQ. These data are in line with the literature both in the CVI [18] and other patients [40] who had begun a guided adapted physical activities program.

The major shortcoming of this study is that the postural outcomes (lordotic angle, pelvic tilting, and pelvic torsion) were not matched at baseline. However, this study was primarily aimed at assessing the efficacy of AS on venous function and quality of life. A future perspective to plan a larger study matching patients with the same postural defects is encouraged. Other limitations are the small study population size and the lack of blindness for the assessors.

## 5. Conclusions

In conclusion, the proposed AS exercise program demonstrated a method by which to improve venous related signs and symptoms, while positively impacting venous drainage function, in correlation with musculoskeletal pump efficiency. Moreover, the amelioration of postural outcomes indicate that the patients received an appropriate treatment for their lower back and pelvis misalignments.

This study paves the way for an increasing clinical role of proper adapted physical activity in the place of management of CVI, which is one of the commonly encountered diseases.

## Figures and Tables

**Table 1:** Active stretching exercise protocol.

**PHASE 1: RESPIRATORY EXERCISES**			
**Exercise Type**	**Starting Position**	**Exercise Performance**	**Exercise Duration**
Upper thoracic respiration (Figure 1A)	Supine position with legs bent and hands resting on the high chest	Breath-in to expand the upper chest ribs towards the hands. Breath-out completely emptying the chest	1′
Diaphragmatic breathing (Figure 1B)	Supine position with legs bent and hands resting at the level of the navel	Deep breath-in, expanding the abdomen and bringing the navel as high as possible. Breath-out slowly, trying to deflate the abdomen as much as possible.	1′
**PHASE 2: VERTEBRAL COLUMN AND PELVIS MOBILIZATION**			
**Exercise Type**	**Starting Position**	**Exercise Performance**	**Exercise Duration**
Antero-retropulsion of the cervical column tract (Figure 1C,D)	Supine position with legs bent and arms to the sides with palms up.	Deep inspiration bringing the chin forward and upwards. Expiration pushing the chin down, trying to flatten the cervical column tract.	1′
Antero and retroversion of the lumbar tract (Figure 1E,F)	Supine position with legs bent and arms to the sides with palms up.	Deep inspiration bringing the pelvis forward against gravity and expiration pushing the pelvis and lumbar tract toward the floor.	1′
**PHASE 3: GLOBAL STRETCHING AND CALF PUMP ACTIVATION**			
**Exercise Type**	**Starting Position**	**Exercise Performance**	**Exercise Duration**
Posterior kinetic chain (Figure 1G,H)	Supine position with legs bent and arms to the sides with palms up.	Raise the arms above the head and bring the right knee to the chest. Coordinate inspiration and expiration phase, detaching the shoulders from the floor and pushing the knee to the chest. Repeat with contralateral leg.	3′
Posterior kinetic chain (Figure 1I,J)	Supine position with legs bent and arms to the sides with palms up.	Bring the knees to the chest. From this position lift the legs up, alternating feet dorsiflexion and plantarflexion.	5′
Posterior kinetic chain (Figure 1K,L)	Supine position with legs lying on the ground and arms to the sides with palms up.	Bring the right knee to the chest grabbing it with the hands. Coordinate inspiration and expiration phase pushing the knee to the chest. Repeat with contralateral leg.	3′
Posterior kinetic chain (Figure 1M)	Supine position with legs bent and arms to the sides with palms up.	Bring the knees to the chest grabbing them with the hands. Coordinate inspiration and expiration phase pushing the knee to the chest and alternating feet dorsiflexion and plantarflexion.	5′
Posterior kinetic chain (Figure 1N,O)	Supine position, lower limbs raised from the ground, flexed leaning against the wall, abducted arms at 120°	Extend right limb with the foot in dorsiflexion, sliding with the heel to the wall upwards. Coordinate inspiration and expiration phase pushing the knee to the chest and alternating feet dorsiflexion and plantarflexion. Repeat with contralateral leg.	5′
**PHASE 4: “CORE” AND LOWER LIMBS STRENGTHENING**			
**Exercise Type**	**Starting Position**	**Exercise Performance**	**Exercise Duration**
Core (Figure 1P)	Quadruped supine position	Extend right leg and bring up the contralateral arm during expiration. Stay in this position for three complete respirations. Repeat with contralateral	3′
Core (Figure 1Q,R)	Sitting position keeping the feet flat on the floor. Abducted arms.	Breath-in and breath-out bringing the right knee close to the chest. Stay in this position for three complete respirations. Repeat with contralateral	3′
Lower limbs strength (Figure 1S,T)	Lateral decubitus legs bent and 1 arm extended	Breath-out lifting the upper leg. Stay in this position for three complete respirations. Repeat with contralateral	3′
Lower limbs strength (Figure 1U,V)	Lateral decubitus with legs extended.	Breath-out lifting the superior leg. Stay in this position for three complete respirations. Repeat with contralateral	3′
**PHASE 5: RELAXATION**			
**Exercise Type**	**Starting Position**	**Exercise Performance**	**Exercise Duration**
Cervical column tract (Figure 1W)	Sitting position	Neck semi-circling	1′
Shoulder (Figure 1X)	Sitting position	Shoulder semi-circling and circling	2′

**Table 2:** Baseline clinical and demographics characteristics of the study population.

	AGS Exercise Group (10 Patients)	Control Group (10 Patients)	*p*
Age (years)	62.9 ± 9.7	54.5 ± 15.5	0.1632
Gender (number of males vs. female)	0 out of 10	2 out of 8	0.9999
BMI	23.3 ± 1.7	24.8 ± 1.7	0.2190
LEG VOLUME (mL)	2340 ± 239	2419 ± 335	0.5496
CEAP	2.7 ± 0.5	2.8 ± 0.4	0.7481
MAIN SYMTOMPS			
Heaviness (number of patients out of total)	10 out of 10	10 out of 10	0.9999
Pain (number of patients out of total)	10 out of 10	10 out of 10	0.9999
Swelling (number of patients out of total)	10 out of 10	10 out of 10	0.9999
Throbbing (number of patients out of total)	7 out of 10	8 out of 10	0.9999
Itching (number of patients out of total)	6 out of 10	7 out of 10	0.9999

BMI: body mass index, CEAP: clinical classification for venous insufficiency [3]. A question regarding the main symptoms was asked of the patients to tell if specific and characteristic symptoms of CVI (heaviness, pain, swelling, throbbing, itching) were present or not.

**Table 3:** Changes in postural parameters from the baseline (T0) to three months follow-up (T3).

	AS Exercise Group			C Group			Reference Value Reported in Literature [16]
	T0	T3	*p*	T0	T3	*p*	
Lordotic angle ITL-ILS max°	51.7 ± 10.5	46.6 ± 9.2	<0.01	39.3 ± 14.8	39.6 ± 14.9	0.6043	35.45 ± 7.55
Pelvic Torsion (DL-DR°)	4.4 ± 2.2	2.5 ± 1.9	<0.01	2.1 ± 2.0	2.0 ± 2.2	0.3434	−0.07 ± 2.95
Pelvic Tilting (DL-DR mm)	5.3 ± 3.6	3.6 ± 2.8	<0.04	6.3 ± 5.1	6.1 ± 5.6	0.3434	−0.12 ± 5.13

ITL: thoraco-lumbar inflexion point, ILS: lumbo-sacral inflexion point, DL: left dimple, DR: right dimple.

## Data Availability

The data that support the findings of this study are available from the corresponding author [S.M.] upon reasonable request.
